# The Singapore Inpatient Psychiatric Rehabilitation Experience

**DOI:** 10.1192/j.eurpsy.2025.2183

**Published:** 2025-08-26

**Authors:** T. Teoh

**Affiliations:** 1 Rehabilitation Service, Institute of Mental Health, Singapore, Singapore

## Abstract

**Introduction:**

Stepping Stones Rehabilitation Centre(SSRC) is a one-year old psychiatric rehabilitation centre in the Institute of Mental Health Singapore.

SSRC was initially created as a 100-bed inpatient ward to address a treatment gap and fast-track the psychiatric rehabilitation of patients admitted for acute psychiatric conditions. SSRC serves as the ‘stepping stone’ for acutely unwell inpatients as they transcend the hospital back to the community. To do so, SSRC actively engages our upstream partners (teams managing acutely unwell patients) as well as downstream partners (social service agencies) to continually assess the treatment gap between acute and community psychiatric care and evolve our programme and processes to close this gap and smoothen the transition.

**Objectives:**

The overall objective is to provide patients with the relevant levels of rehabilitation by training and equiping them with the necessary skills to intergrate back to society at the level for which they have set their goals on.The levels of rehabiliatation range from intermediate rehabilitation following acute illness to regain their functional status to perform their daily activities to tertiary rehabilitation which provides higher intensity rehabilitation programmes to help patients return to their daily function and to work and life as much as possible. This is achieved via a multi-disciplinary team comprising of patient, psychiatrists, doctors, nurses, occupational therapists (OT), medical social workers (MSW), psychologists, peer support specialists (PSS) and case managers(CM) to derive a bespoke rehabilitation programme for each patient over their course of stay in the ward.

**Methods:**

Patient are referred to the SSRC from the acute inpatient wards of IMH or from the outpatient setting. Upon acceptance to the ward, patients are reviewed and a timetable is created based on the goals the patient would like to achieve. Patients are reviewed at weekly MDT (multi-disciplinary team) meetings at frequencies which commensurate to their rate and intensity of rehabilitation. Measures obtained to assess the progress of each patient and the programme are:

1. CGI-I (Clinical Global Impression - improvement) scale
2. GAF (Global Assessment of Functioning)
3. RAS-DS (Recovery Assessment Scale - Domains and Stages)
4. SLOF (Specific Level of Functioning)
5. Acceptance rates to stepdown care
6. 30-day readmission rates

**Results:**

At the latest tabulation of data, there has been 117 patients successfully discharged from SSRC. The other data are currently being tabulated and analysed and updated results will be shared at the meeting.

**Conclusions:**

SSRC is one of few psychiatric rehabiliatation entities that reside in an acute psychiatric hospital. It was assessed that patients who present with acute decompensation in their mental state could benefit from intense fast-tracked rehabilitation measures before stepping down to community provided services.

**Disclosure of Interest:**

None Declared

